# Anti-glycation, antiplatelet and antioxidant effects of different pomegranate parts

**DOI:** 10.1186/s12906-022-03824-6

**Published:** 2022-12-27

**Authors:** Zahra Amri, Ikram Ben Amor, Amira Zarrouk, Raja Chaaba, Jalel Gargouri, Mohamed Hammami, Sonia Hammami

**Affiliations:** 1 Biochemistry Laboratory, LR12ES05 “Nutrition- Functional Foods and vascular Health”, Faculty of Medicine, University of Monastir, 5019 Monastir, Tunisia; 2 Centre Régional de Transfusion Sanguine de Sfax, Route El-Ain Km 0.5, CP 3003 Sfax, Tunisia

**Keywords:** Pomegranate, Antiplatelet activity, Advanced glycation end-products, Oxidative stress, Lipid peroxidation

## Abstract

**Background:**

Platelet aggregation and advanced glycation end products (AGEs) and oxidative stress are known as key factors for the development of cardiovascular diseases and diabetic complications. In this context, fruit and vegetable consumption, good sources of antioxidant compounds have been largely reported as an effective way of preventing human against these diseases. The current study focuses on the evaluation of antioxidant, antiplatelet and anti-glycation activities of pomegranate (*Punica granatum L.*) flowers (PF), leaves (PL), peel (PP) juice (PJ) and seeds oil (PSO).

**Methods:**

Antioxidant activities was measured against ABTS radical and lipid peroxidation. Antiglycation activity was determined using the formation of AGE fluorescence intensity in the BSA/ribose system. Antiplatelet activity was measured in platelet rich plasma (PRP) against adenosine diphosphate (ADP), Collagen and arachidonic acid (AA).

**Results:**

PF extract displayed the highest antioxidant activity against ABTS and lipid peroxidation with IC_50_ values of 0.7 mg/mL and 0.63 mg/mL respectively. For anti-glycation activity, PP, PF and PL inhibited moderately the pentosidine-like AGEs formation compared to positive controls with AGE-IC_50_ value of 0.4 mg/mL. PJ and PSO haven’t any anti-AGE effect. All the extracts selectively inhibited platelet aggregation caused by one, two or three inducers in dose dependent manner. PF was the most potent inhibitor caused by all three inducers, with inhibitory effects ranging from 35.6 to 66.6%. PP and PJ exhibited antiplatelet effect against both ADP and collagen and PL and PSO only against AA.

**Conclusions:**

These results suggest that some pomegranate extracts exert potential in vitro anti-glycative and antiplatelet activities.

## Background

Pomegranate (*Punica granatum L.*), has been widely known as a very potent antioxidant fruit [1–3]. The antioxidant power of pomegranate juice has been reported to be 3-fold higher than that of red wine or green tea [4] and 8-fold higher levels than those detected in orange juices [5]. In addition, one natural fruit that is under much research is the pomegranate and its constituents which have been reported to have strong biological activity and medicinal value. In fact, pomegranate juice, peel, seeds oil, leaves and flower extracts have been described to have in vitro as well as in vivo antidiabetic [6], anti-inflammatory [7], antioxidant, anti-obesity [8] and anti-tumor effects [9]. These beneficial effects are related to the presence of very high levels of antioxidants such as polyphenolic compounds, including hydrolysable tannins, anthocyanins and flavonols [10]. In our previous studies on the antidiabetic effects of pomegranate, results highlight the neuroprotective effects of pomegranate extracts and demonstrate that a long-term intake of pomegranate might be a potential alternative strategy for the prevention of an HFD (High Fat High Fructose Diet) induced insulin resistance and oxidative stress [6, 11]. In fact, pomegranate juice, leaves and peel consumption resulted in a significant reduction in fasted plasma glucose and insulin levels. Consequently, the homeostatic index of insulin resistance (HOMA-IR) which is used to quantify insulin resistance was respectively reduced indicating a significant improvement in insulin sensitivity.

In this context, we made an attempt to evaluate the effect of pomegranate extracts against the most known diabetes complications such as platelet aggregation and Advanced glycation end products (AGEs) which have been reported to be correlated with the progression of diabetes and aging [12, 13]. In fact, the inhibition of platelet function has been adopted for a long time as a strategy to treat acute vascular atherothrombotic diseases such as diabetes cardiovascular diseases and ischemic stroke [14, 15]. Advanced glycation end products (AGEs) are associated with greater risk of diabetic complications such as diabetic retinopathy, neuropathy, and nephropathy [16]. In addition, few reports have been issued on the inhibitory effect of different pomegranate tree parts on AGE formation [17] or platelet aggregation [18]. In this work, we investigated in vitro the anti-AGE and antiplatelet capacities and some antioxidant activities of pomegranate juice (PJ), peel (PP), flowers (PF), leaves (PL) and seeds oil (PSO).

## Methods

### Plant materials and extraction

Leaves and fruits were harvested from *Tounsi* pomegranate trees in October 2021 from Mahdia region, Tunisia. Variety authenticity was confirmed by taxonomist Dr. Faten Zaouay from the Department of Horticulture, Higher Agronomic Institute, Chott-Meriem (University of Sousse, Tunisia) and a voucher specimen was deposited in our national collection maintained in duplicate at Gabes and Chott-Mariem (Sousse), with the code ‘TNl, TN2, TN3, TN5, TN5”.

Pomegranate extracts were prepared as described by our previous study [11]. Fruits were washed and hand-peeled. Arils were squeezed using a commercial blender (moulinex, France). The extract juice was centrifuged at 15000 rpm for 15 min. Then the supernatant was recuperated and lyophilized. Leaves, flowers and fruit peel were dried, powdered and extracted with methanol (MeOH) 50 g/250 ml in the dark for 48 hours. Each extract was filtered through Whatman No. 42 filter paper and evaporated to dryness using a rotary evaporator (Heidolph, Germany) under vacuum at 45 °C and stored at − 20 °C for further determination. Pomegranate seeds were dried and powdered. Oil was extracted by the methods of soxhlet. About 30 g seeds were extracted with 200 ml of hexane at room temperature for 6 h. The solvent was removed by evaporation at 40 °C and the oil was flushed with nitrogen stream and stored at − 20 °C in sealed tubes.

### ABTS radical scavenging assay

The antioxidant capacity of pomegranate extracts by the ABTS (2,2′-azino-bis (3-ethylbenzothiazoline-6-sulfonic acid) assay was measured using a previous method [19]. Briefly, ABTS• + radical solution was produced by reacting the ABTS stock solution (5 mM) with potassium persulfate (K_2_S_2_O_8_) solution (2.7 mM). For the evaluation of antioxidant capacity, the ABTS• + solution was diluted with phosphate buffer (20 mM, pH 7.4) to obtain the absorbance of 0.700 ± 0.020 at 660 nm. Then, ABTS• + solution was mixed with pomegranate extracts prepared at different concentrations. After incubation, the absorbance was measured at 734 nm. Ascorbic acid was used as the positive control. The percentage of inhibition of ABTS• + radical was calculated with the following formula:

$$\textrm{Inhibition}\;\left(\%\right)=\left[\left(\textrm{A}\ \textrm{control}-\textrm{A}\ \textrm{sample}\right)/\textrm{Acontrol}\right]\ast 100$$

Acontrol refers to the solution containing pure MeOH instead of the sample, and Asample refers to the absorbance of pomegranate extract containing solutions. The effective concentration of sample necessary to decrease the absorbance ABTS• + by 50% (EC_50_) was determined.

### Lipid peroxidation using ferric thiocyanate method

Inhibition of lipid peroxidation by pomegranate extracts was assayed according the previous procedure [20]. Linoleic acid (LA) was used as the lipid matrix and 2,2′-azobis (2-methylpropionamidine) dihydrochloride (AAPH) as the free radical initiator. Different concentrations of each pomegranate extract were prepared. Each concentration was mixed with 1.3% (w/v) methanolic LA and 0.2 M phosphate buffer (pH 7.0) and the peroxidation was initiated by the addition of AAPH solution (55.3 mM) in phosphate buffer. The control solution was prepared by adding pure MeOH instead of the sample. After incubation at 50 °C for 24 h in the darkness, the reaction mixture was dissolved in a 3:1 (v/v) H_2_O–MeOH solution. Then, a 10% aqueous solution of NH_4_SCN and 20 mM FeCl_2_ in 3.5% HCl were added. After 3 min of incubation at room temperature, the absorbance was measured at 546 nm against the corresponding blank. Ascorbic acid was used as the positive control. The results are expressed as the percentage of lipid peroxidation inhibition:

$$\%\textrm{Inhibition}=\left(\textrm{Acontrol}-\textrm{Asample}\right)\ast 100/\textrm{Acontrol}$$

Acontrol refers to the solution containing pure MeOH instead of the sample, and Asample refers to the absorbance of oil-containing solutions. The EC_50_ was determined.

### Advanced glycation end-products inhibition assay

Inhibition of pentosidine-like AGEs formation and EC_50_ values were determined and calculated using a previously described method by Séro et al. 2013, with slight modifications [21]. Briefly, BSA (10 mg/mL) was incubated with D-ribose (0.5 M) together with the tested extract in 50 mM phosphate buffer at pH 7.4 (NaN_3_, 0.02%). Solutions were incubated in 96-well microtiter plates at 37 °C for 24 h in a closed system before AGE fluorescence measurement. Fluorescence resulting from the incubation, under the same BSA (10 mg/mL) and tested extract conditions, was subtracted for each measurement. Pentosidine-like (λexc 335 nm, λem 385 nm) AGEs fluorescence was measured using a microplate spectrofluorometer. The percentage of AGEs formation was calculated as follows for each extract concentration and the EC_50_ values were determined:

$${\displaystyle \begin{array}{l}\textrm{AGEs}\;\left(\%\right)=\left[\textrm{fluorescence}\ \textrm{intensity}\;\left(\textrm{sample}\right)-\textrm{fluorescence}\ \textrm{intensity}\;\left(\textrm{blank}\ \textrm{of}\ \textrm{sample}\right)\right]\\ {}{}^{\ast }100/\left[\textrm{fluorescence}\ \textrm{intensity}\ \left(\textrm{control}\right)-\textrm{fluorescence}\ \textrm{intensity}\left(\textrm{blank}\ \textrm{of}\ \textrm{control}\right)\right]\end{array}}$$

### In vitro evaluation of anti-platelet aggregation activity

Fresh blood was obtained from healthy volunteers with negative history of consumption of drug, beverages or foods that may affect aggregation for at least 10 days and preferably should have fasted overnight because the presence of chylomicron may also disturb the aggregation patterns. The study was approved by the local ethics committee of the University Hospital Hedi Chaker of Sfax, Tunisia.

Venous blood was collected in a plastic tube containing trisodium citrate 109 mM. PRP was obtained by centrifuging at room temperature for 12 min at 200×g. PRP was removed carefully avoiding contamination with red cells or buffy coat, and stored at room temperature until tested. All the tests should be completed within 3 hours of preparing the PRP. The remaining blood was than centrifuged at 2000×g for 20 min to obtain platelet-poor plasma (PPP). We used a screening panel of aggregating agents: adenosine 5′-diphosphate (ADP, 20 μM), collagen (5 μg/mL) and arachidonic acid (2 mM).

PRP and PPP were used to set, respectively, 0 and 100% light transmission in the aggregometer. Platelet aggregation was monitored for at least 5 minutes after adding an agonist.

For pomegranate leaves (PL), flowers (PF), juice (PJ) and peel (PP) extracts, different concentrations were prepared previously for each extract dissolved in DMSO (at 0.05% final concentration). For PSO, different concentrations were dissolved in 70% Polyethylene glycol (PEG) which is a widely used solvent in an in vivo to dissolve water-insoluble compounds. Ten microliters of each extract were added to 260 μL of control PRP, and then the mixture was incubated for at least 5 minutes (until 30 min) at 37 °C before adding agonists. Then collagen (5 μg/mL), AA (2 mM) or ADP (20 μmol/L) was added and platelet shape change and aggregation were monitored for 5 min. DMSO (0.5% v/v) was used as negative control and aspirin was used as positive control.

The extent of platelet aggregation was calculated by the following formula:

$$\begin{array}{l}\text{Inhibition}\;\%=\left[1-\left(\text{D}/\text{S}\right)\right]\times100\end{array}$$

D = platelet aggregation in the presence of test compounds

S= platelet aggregation in the presence of solvent.

The platelet aggregation inhibitory activity was expressed as percent inhibition by comparison with that measured for the vehicle (DMSO or PEG) alone. Each sample was measured in triplicate and the data are presented as mean ± SD. The values of effective concentrations required for 50% inhibition of platelet aggregation (EC_50_), were obtained from at least three determinations.

### Statistical analysis

Results were expressed as the mean of at least three independent measurements, unless standard deviations have been reported (means ± SD) and analyzed using SPSS ver. 21.0, professional edition. For antioxidant activities, Duncan’s test was used to estimate the significance among the main effects at the 5% probability level (*P* < 0.05).

## Results and discussion

### Antioxidant proprieties of pomegranate extracts

The antioxidant capacities of pomegranate extracts were measured by ABTS and lipid peroxidation assays. The results were summarized in Table 1 and were expressed as the EC_50_ value. Lower EC_50_ indicated higher antioxidant activity. It was found that the extracts differ from one another in term of their antioxidant effectiveness. For instance, PF displayed the highest antioxidant activity against ABTS with an EC_50_ values of 0.7 mg/ml, superior even to the standard ascorbic acid which had an IC50 of 1.4 mg/ml. PF showed the second lowest EC50 for lipid peroxidation assay (0.63 mg/mL), slightly larger than the standard ascorbic acid (0.52 mg/mL), however this difference was not statistically significant (*p* < 0.05). PP extract followed by PL and PJ extracts are able to effectively reduce the free radical ABTS. The same order was found in lipid peroxidation tests. However, PSO demonstrated the weakest antioxidant activity in both in vitro assays. It is reported that there is an established relationship between the phenolic content and the antioxidant capacity [22]. In our previous study [23], we studied the phenolic contents of pomegranate flowers, leaves, peel and juice and we compared their reducing power and anti-DPPH activity. Results show that all organs had also an effective reducing power and antiradical activity. Flowers and leaves were richer in phenols and proved to be the strongest antioxidants.

**Table 1:** Antioxidant capacities of different pomegranate extracts

	ABTS (EC_**50**_ mg/mL)	Lipid peroxidation inhibition (EC_**50**_ mg/mL)
Ascorbic acid	1.4 ± 0.2^b^	0.52 ± 0.14^a^
PP	1.5 ± 0.08^b^	2.1 ± 0.05^c^
PF	0.7 ± 0.01^a^	0.63 ± 0.13^a^
PL	2.2 ± 0.2^c^	1.5 ± 0.2^b^
PJ	2.2 ± 0.15^c^	5.1 ± 0.4^d^
PSO	3.0 ± 0.3^d^	5.4 ± 0.15^d^

^a-d^ different small letters in the same column indicate significant statistical differences (Duncan’s Test. *p* < 0.05) among extracts. Ascorbic acid: positive control; *PP* pomegranate peel; *PF* pomegranate flowers; *PL* pomegranate leaves; *PJ* pomegranate Juice; *PSO* Pomegranate Seeds Oil

### Anti-AGEs capacities of pomegranate extracts

**Fig. 1:**
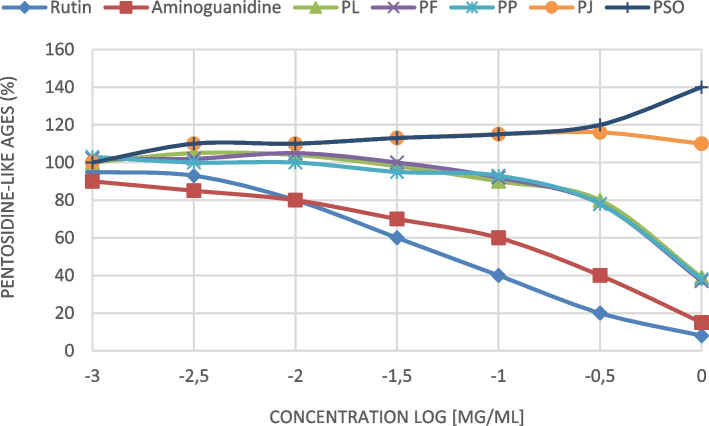
Dose-effect curves for pentosidine-like AGE formation in the presence of pomegranate extracts. PP: pomegranate peel; PF: pomegranate flowers; PL: pomegranate leaves; PJ: pomegranate Juice; PSO: Pomegranate Seeds Oil

The anti-glycation capacities of pomegranate extracts evaluated by their inhibition of the formation of global fluorescent AGEs in the BSA/ribose system are depicted in Fig. 1 and Table 2. PP, PF and PL extracts demonstrated a dose-response inhibition of the pentosidine-like AGEs formation (Fig. 1) with AGE-EC_50_ value of 0.4 mg/ml (Table 2). This anti-AGEs capacity is considered moderate compared to that exhibited by Aminoguanidine (AGE-EC_50_; 0.16-0.17 mg/mL) and weak compared to Rutoside trihydrate (AGE-EC_50_; 0.05 mg/mL). However, results show that PJ and PSO haven’t any anti-AGE effect (AGE-EC_50_; > 1 mg/mL). In double blind study, Sohrab (2015) concluded that pomegranate *(Punica granatum)* juice decreases lipid peroxidation, but has no effect on plasma advanced glycated end-products in adults with type 2 diabetes [24]. Our results concerning pomegranate juice are not in line with some past findings reported by Liu (2014), who founds that pomegranate fruit extract (PFE) showed potent anti-glycation activity [25]. The anti-glycation activity of different pomegranate extracts can be attributed to its phenolic constituents. In fact, Kumagai (2015), showed that the AGEs formation derived from BSA with glucose, fructose, and glyceraldehyde in vitro was concentration-dependently suppressed by addition of pomegranate fruit extract PFE and its phenolic components such as punicalin, punicalagin, ellagic acid, and gallic acid [17].

**Table 2:** Effect of different pomegranate extracts on pentosidine-like age formation expressed as EC_50_ (mg/mL)

Extract	Quantity (mg)	Molecular weight (g.mol-1)	EC_**50**_ pentosidine-like ages mm (molecules) or mg/mL (extract)
Aminoguanidine^a^		110	0.16-0.17 mg/mL / 1.4-1.5 mM
Rutoside trihydrate^a^		664	0.05 mg/mL / 75 μM
PL	2.15		0.4 mg/mL
PF	3.90		0.4 mg/mL
PSO	3.28		> 1 mg/mL
PP	2.20		0.4 mg/mL
PJ	2.35		> 1 mg/mL

^a^ positive control; *PP* pomegranate peel; *PF* pomegranate flowers; *PL* pomegranate leaves; *PJ* pomegranate Juice; *PSO* Pomegranate Seeds Oil

### Antiplatelet activity of pomegranate extracts

Pomegranate parts were evaluated for their ability to inhibit platelet aggregation of human PRP induced by ADP, Collagen and AA as potent aggregation inducers. Table 3 shows the inhibitory effects of different extracts at various concentrations and aspirin as positive control and Table 4 summarized the EC_50_ values of pomegranate extracts or compounds with the mean values of three measurements. All the extracts selectively inhibited platelet aggregation caused by one, two or three inducers in dose dependent manner.

**Table 3:** Percentage inhibition of different pomegranate extracts and some of their compounds on platelet aggregation in human whole blood induced by and adenosine diphosphate (ADP). collagen and arachidonic acid (AA)

Extract or compound	Concentration (mg/mL)	ADP (20 μM)	Collagen (5 μg/mL)	AA (2 mM)
PP	50	43.6 ± 3.2	52.6 ± 2.5	–
	25	29 ± 3.6	21 ± 1.7	–
PF	3.5	35.6 ± 4	66.6 ± 3.5	45.6 ± 2
	1.75	26.6 ± 2.8	44.3 ± 2.5	13.6 ± 2.1
	1	9 ± 1	–	7.6 ± 2.5
PL	50	16.6 ± 1.5	–	–
	10	–	–	–
PJ	150	53.3 ± 2.8	32.3 ± 2.5	–
	75	22 ± 2	18.3 ± 1.5	–
PSO	50	46.3 ± 0.5	–	–
	25	9 ± 1.7	–	–
	1	–	–	–
Aspirin	75	45.2 ± 1	–	100 ± 0.1

(−) No effect; Aspirin: positive control; *PP* pomegranate peel; *PF* pomegranate flowers; *PL* pomegranate leaves; *PJ* pomegranate Juice; *PSO* Pomegranate Seeds Oil

**Table 4:** EC_50_ values (mg/ml) of different pomegranate extracts on platelet aggregation induced by three different aggregating agents

	ADP	Collagen	AA
PP	57.46 ± 4.39^c^	47.54 ± .29	–
PF	4.86 ± 0.68^b^	2.8 ± 0.06	3.85 ± 0.19^b^
PL	150.87 ± 14.27^d^	–	–
PJ	164.54 ± 15.02^d^	197.25 ± 16.05	–
PSO	53.96 ± 0.66^c^	–	–
Aspirin	0.42 ± 0.04^a^	–	0.66 ± 0.03^a^

(−) No effect. Concentrations of agonists were as follows: collagen (5 μg/mL). arachidonic acid (AA; 2 mM). adenosine diphosphate (ADP; 20 μmol/L). *Aspirin* positive control; *PP* pomegranate peel; *PF* pomegranate flowers; *PL* pomegranate leaves; *PJ* pomegranate Juice; *PSO* Pomegranate Seeds Oil

Flowers extract was found to be the most potent inhibitor of platelet aggregation caused by all three inducers, with inhibitory effects ranging from 35.6 to 66.6% at 3.5 mg/mL. In fact, it was active against collagen-induced platelet aggregation with an EC_50_ value of 2.8 mg/mL, then against AA-induced platelet aggregation with an EC_50_ value of 3.85 mg/mL and with 4.86 mg/mL when aggregation was stimulated by ADP. Compared to Aspirin as positive control, PF, PP and PJ have inhibitory effect against aggregation induced by collagen. However, Aspirin inhibited aggregation induced by AA and ADP with as EC_50_ of 0.42 and 0.66 mg/ml respectively but no effect was found against collagen. In this study and in our previous study [23], PF are found to be the most antioxidant pomegranate part against DPPH radical, ABTS radical and lipid peroxidation comparing to PP, PL and PJ. This finding may be explaining that’s why PF was the best inhibitor of platelet aggregation. In addition, PF are rich in phenols (16.6%) including mainly hydrolyzed tannins (ellagitannin) and in soluble dietary fiber (30.2%) [26]. Hydrolyzed tannins have been previously demonstrated to be very effective in inhibiting platelet function [18]. On the other hand, the antiplatelet activity of dietary fiber was wet uncertain [27, 28]. So, we hypothesized that the potent and multi-targeted antiplatelet activity of PF can be attributed to hydrolyzed tannins, major phenols found in this organ.

PP and PJ exhibited inhibitor effect against both ADP and collagen-induced platelet aggregation. However, no effect was shown for both extracts when AA was used as agonist. Our results do not confirm with that found by Mattiello et al., 2009 who show that both extracts inhibit platelet response to AA [18]. The comparison of EC_50_ values revealed that PP decreased ADP and collagen-induced platelet aggregation more efficiently than PJ.

The difference in inhibitory effect between both extracts can be explained by the difference in antioxidant capacity. PP was more potent antioxidant than PJ against ABTS radical and lipid peroxidation in this study and also against DPPH radical and reducing power [23]. This explanation was in disaccord with some previous reports which suggested that the antiplatelet potential of fruits appeared to be unrelated or opposite to their antioxidant activity [29, 30]. PL and PSO were able to inhibit just ADP-triggered platelet aggregation whereas they were no effective when collagen and AA were used as agonists.

## Conclusion

In conclusion, pomegranate flowers, leaves and peel have in vitro inhibitory effects on protein glycation and platelet aggregation. These effects were attributed to the antioxidant properties of several pomegranate active compounds. However, further research is necessary to confirm these results and to obtain a deeper understanding of its mechanism of action, before being proposed as a natural AGE inhibitor. The antioxidant property? Active compounds in pomegranate that potentially/ contribute to these properties.

## Data Availability

The datasets generated during and/or analyzed during the current study are available from the corresponding author on reasonable request.

## Abbreviations

AA: arachidonic acid
AAPH: 2,2′-azobis (2-methylpropionamidine) dihydrochloride
ABTS: 2,2′-azino-bis (3-ethylbenzothiazoline-6-sulfonic acid
ADP: Adenosine Diphosphate (ADP)
AGEs: Advanced Glycation end products
BSA: Bovin Serum Albumin
DMSO: Dimethyl Sulfoxide
FeCl_2_: Ferrous chloride
HCl: Chlorhydric acid
HFD: High Fat High Fructose Diet
K_2_S_2_O_8_: Potassium persulfate solution
LA: Linoleic acid
MeOH: Methanol
NaN_3_: Sodium azide
NH_4_SCN: Ammonium thiocyanate
PEG: Polyethylene glycol
PRP: Platelet rich plasma
PPP: Platelet poor plasma
